# From orthopedics to oral and maxillofacial surgery: extension and insights of mesenchymal stem cell-based therapeutic strategies

**DOI:** 10.1097/JS9.0000000000003348

**Published:** 2025-09-09

**Authors:** Yiming Ji, Shichen Lin

**Affiliations:** Department of Stomatology, Electric Power Teaching Hospital, Capital Medical University, Beijing, China

*Dear Editor,*

We have carefully reviewed the article *“Recent Trends of Stem Cell Therapies in the Management of Orthopedic Surgical Challenges”* by Pal et al., recently published in the *International Journal of Surgery* (2024)^[1]^. The authors systematically summarized recent advances in stem cell applications in orthopedic surgery. However, the article pays limited attention to bone defects in the oral and maxillofacial region, which are structurally complex, biomechanically demanding, and inherently challenging to regenerate. Therefore, we would like to provide additional commentary, particularly by integrating recent progress in oral and maxillofacial tissue regeneration and highlighting the unique value of mesenchymal stem cells (MSCs) in this field.

## Oral and maxillofacial bone defects: etiologies and clinical challenges

Bone defects in the oral and maxillofacial region can result from a variety of causes, including trauma, infections, cystic lesions, tumor resection, and congenital anomalies. Among these, periodontitis is a common cause of alveolar bone loss and tooth extraction, posing a pressing need for effective bone regeneration^[2]^. Furthermore, surgical resection of benign or malignant tumors often leads to large segmental defects in the mandible or maxilla. Conventional autologous bone grafts are associated with donor site morbidity and unpredictable outcomes, prompting the development of stem cell-based tissue engineering approaches^[1]^.

## Common MSC sources in the oral and maxillofacial region

Due to their multipotent differentiation capacity, immunomodulatory effects, and angiogenic support, MSCs are widely applied in tissue regeneration. The oral region offers several accessible sources for MSC harvesting, including alveolar bone marrow, dental pulp, periodontal ligament, and gingival tissues. Among them, alveolar bone marrow-derived MSCs (aBMSCs) have gained increasing attention due to their anatomical proximity to defect sites and relative ease of harvesting.

Mason et al. established a standardized procedure for the isolation and expansion of aBMSCs, demonstrating their favorable multilineage potential, biosafety, and osteogenic capability, thus laying a foundation for clinical-scale application^[3]^. In addition, dental-derived MSCs – such as dental pulp stem cells (DPSCs) and periodontal ligament stem cells (PDLSCs) – have shown promising results in regenerating periodontal tissues and other soft-hard oral structures^[4]^.

## Supplementary insights into mesenchymal stem cell applications in oral and maxillofacial surgery

Although Pal *et al* provided a comprehensive summary of MSC applications in orthopedics, the indications and specific advantages of MSCs in oral and maxillofacial surgery warrant further discussion. We believe the following aspects are particularly important:

Firstly, MSCs derived from the oral region are more clinically accessible. For instance, aBMSCs can be harvested relatively easily, and their anatomical origin matches the defect site, potentially enhancing tissue integration and osteoinductive outcomes^[3]^.

Secondly, the osteogenic potential of MSCs is influenced by several regulatory mechanisms, among which epigenetic modulation has recently gained attention. Kulterer *et al* reported that modifying the epigenetic state of jaw periosteal cells – such as through histone acetylation – can significantly enhance their osteogenic differentiation, offering a new strategy for precise MSC application^[5]^.

Thirdly, standardization in the preparation and expansion of MSCs is essential for clinical translation. Kaigler’s team proposed a GMP-compatible workflow for the isolation, expansion, and quality control of aBMSCs, providing a reliable protocol for clinical-scale production^[3]^.

Lastly, the choice of delivery scaffold significantly influences the regenerative outcome of MSCs. Zhu *et al* utilized NELL-1-modified MSCs combined with PLGA scaffolds in a goat mandibular condyle cartilage defect model, achieving simultaneous regeneration of cartilage and subchondral bone with favorable outcomes^[6]^. Moreover, decellularized tissue matrices are increasingly being explored as natural scaffolds for periodontal regeneration^[7]^.

## Conclusion

In summary, the review by Pal *et al* offers valuable insight into the frontier of MSC application in orthopedic surgery. We suggest that future reviews and studies place more emphasis on the distinctive features and challenges of MSC-based therapies in oral and maxillofacial surgery. Local MSC sources such as aBMSCs and dental-derived MSCs, when combined with epigenetic enhancement and advanced biomaterial-based delivery systems, present promising prospects. Standardizing cell sourcing, expansion protocols, and scaffold systems will be crucial for realizing the clinical translation of MSC therapies in oral and maxillofacial surgery.

This manuscript was entirely prepared by the authors without the use of any AI tools, in accordance with the transparency requirements outlined in the TITAN reporting checklist^[8]^.

## Key learning points

1. Oral and maxillofacial bone defects present unique clinical challenges

Compared with general orthopedic defects, oral and maxillofacial defects are more structurally complex and biomechanically demanding, often resulting from periodontitis, trauma, or tumor resection.

Local MSC sources are more clinically accessible and site-specific

Alveolar bone marrow-derived MSCs (aBMSCs) and dental-derived MSCs (e.g., DPSCs, PDLSCs) are anatomically relevant and easier to harvest, offering enhanced compatibility with defect sites.

Epigenetic regulation enhances osteogenic differentiation of MSCs

Histone modification and other epigenetic interventions can significantly improve the osteogenic potential of MSCs, providing a promising strategy for precision regenerative therapy.

Standardized GMP-compatible protocols are essential for clinical translation

Reliable workflows for MSC isolation, expansion, and quality control are critical to ensuring reproducibility and safety in clinical applications.

Scaffold selection directly impacts MSC-based regeneration outcomes

Biomaterial scaffolds, such as PLGA or decellularized matrices, play a key role in guiding MSC differentiation and enhancing bone and periodontal tissue regeneration.

Future research should focus on integration of stem cell sources, epigenetic tools, and biomaterial systems

A synergistic approach combining local MSCs, molecular modulation, and optimized delivery platforms holds promise for advancing regenerative strategies in oral and maxillofacial surgery.

## Data Availability

No new data were generated or analyzed in this study.
